# Mechanism of Dyspnea during Exercise in Children with Corrected Congenital Heart Disease

**DOI:** 10.3390/ijerph19010099

**Published:** 2021-12-23

**Authors:** Mehdi Chlif, Mohamed Mustapha Ammar, Noureddine Ben Said, Levushkin Sergey, Said Ahmaidi, Fawaz Alassery, Habib Hamam

**Affiliations:** 1 EA 3300 “APS and Motor Patterns: Adaptations-Rehabilitation”, Picardie Jules Verne University, 80025 Amiens, France; 2 National Center of Medicine and Science in Sports (NCMSS), Tunisian Research Laboratory Sports Performance Optimization, Ave Med Ali Akid, El Menzah, Tunis 263, Tunisia; said.ahmaidi@gmail.com; 3 Exercise Physiology Department, College of Sport Sciences and Physical Activity, King Saud University, C.P. 22480, Riyadh 11495, Saudi Arabia; dr.mohammar@gmail.com; 4 Department of Biomechanics and Motor Behavior, College of Sport Sciences and Physical Activity, King Saud University, C.P. 22480, Riyadh 11495, Saudi Arabia; bensaeed@mail.ru; 5 Federal State-Funded Scientific Institution “Institute of Developmental Physiology of the Russian Academy of Education”, Russian State University of Physical Culture, Sport, Youth and Tourism (SCOLIPE), 105122 Moscow, Russia; levushkinsp@mail.ru; 6 Department of Computer Engineering, College of Computers and Information Technology, Taif University, P.O. Box 11099, Taif 21944, Saudi Arabia; falasser@tu.edu.sa; 7 Faculty of Engineering, Moncton University, Moncton, NB E1A 3E9, Canada; Habib.Hamam@umoncton.ca

**Keywords:** dyspnea threshold, ventilatory threshold, congenital heart disease, exercise capacity

## Abstract

This study will evaluate cardiorespiratory and peripheral muscle function and their relationship with subjective dyspnea threshold after the surgical correction of congenital heart disease in children. Thirteen children with surgically repaired congenital heart disease were recruited. Each participant performed an incremental exercise test on a cycle ergometer until exhaustion. Gas exchanges were continuously sampled to measure the maximal aerobic parameters and ventilatory thresholds. The functional capacity of the subjects was assessed with a 6 min walk test. At the end of the exercise test, isokinetic Cybex Norm was used to evaluate the strength and endurance of the knee extensor muscle in the leg. Dyspnea was subjectively scored with a visual analog scale during the last 15 s of each exercise step. Oxygen consumption measured at the dyspnea score/VO_2_ relationship located at the dyspnea threshold, at which dyspnea suddenly increased. Results: The maximal and submaximal values of the parameters describing the exercise and the peripheral muscular performances were: VO_2_ Peak: 33.8 ± 8.9 mL·min^−1^·kg^−1^; HR: 174 ± 9 b·min^−1^; VEmax: 65.68 ± 15.9 L·min^−1^; P max: 117 ± 27 W; maximal voluntary isometric force MVIF: 120.8 ± 41.9 N/m; and time to exhaustion Tlim: 53 ± 21 s. Oxygen consumption measured at the dyspnea threshold was related to VO_2_ Peak (R^2^ = 0.74; *p* < 0.01), Tlim (R^2^ = 0.78; *p* < 0.01), and the distance achieved during the 6MWT (R^2^ = 0.57; *p* < 0.05). Compared to the theoretical maximal values for the power output, VO_2_, and HR, the surgical correction did not repair the exercise performance. After the surgical correction of congenital heart disease, exercise performance was impeded by alterations of the cardiorespiratory function and peripheral local factors. A subjective evaluation of the dyspnea threshold is a reliable criterion that allows the prediction of exercise capacity in subjects suffering from congenital heart disease.

## 1. Introduction

Because of recent improvements in science and technology, as well as more successful cardiovascular disease diagnosis, the number of people living with congenital heart disorders (CHD) and reaching adulthood is increasing [1,2]. Congenital heart disease (CHD) patients have a variety of physiologic issues, including decreased aerobic capacity, exertional dyspnea, cardiovascular and peripheral muscle deconditioning, and muscle atrophy and weakness [3,4,5]. There is earlier evidence that exercise capacity is not regulated by cardiac variables in isolation, but depends on a complex interplay between cardiopulmonary and muscular components in CHD [6,7]. Exercise intolerance, due to dyspnea and fatigue, is a defining feature of CHF [8,9,10]. Previously, these symptoms were thought to be entirely the result of central hemodynamic derangements, which could be reversed with inotropic agents and/or vasodilators. However, it is now clear that CHF has a significant skeletal muscular pathology, which can contribute to the associated symptoms. To identify the underlying physiopathological mechanisms of exercise intolerance, incremental maximal exercises are mostly used [11,12]. The cardiopulmonary exercise test (CPET) assesses overall functional ability, allowing for the determination of disease severity, progression, prognosis, and therapeutic approaches [9]. Thus, the most operated subjects are suffering from exercise limitation, which is a critical determinant of their outcomes [13,14]. However, the usefulness of the assessment of maximal exercise capacity can be questioned, since these subjects are unable to perform maximal exercise during their normal daily life activities.

A reduced functional capacity and exercise tolerance are related to a poor quality of life and a worse prognosis is evident in people with CHD. The 6 minute walking test (6 MWT) is a widely available and well-tolerated tool for assessing the functional capacity in CHD patients. Although the cardiopulmonary exercise test (a maximal exercise test) is still the gold standard for assessing the exercise capacity in CHF patients, the 6MWT (submaximal exercise test) can provide reliable information regarding the patient’s everyday activity.

Frequently, patients complain about dyspnea as a limiting factor [15,16,17,18,19]. Several authors found reduced values of maximal oxygen consumption, power output, and heart rate in children after surgical correction of congenital heart disease [9,18]. Peak VO_2_ correlates poorly with the indices of central hemodynamic functions [20], and attention has turned to the periphery as an explanation for exercise limitation [20,21].

Evidence suggests that that significant skeletal muscular pathology is also present in heart disease and can contribute to exercise intolerance due to dyspnea and fatigue [22]. Muscle performance is mostly determined by strength and endurance. When either of these variables is impaired, muscle weakness and performance are reduced as a result of metabolic abnormalities and the insufficient energy in the muscles. The lower limb skeletal muscles belonging to the subjects become abnormal in heart disease [5]. There is reduced blood flow to the legs during exercise, and function is reduced in terms of both the isometric force production and increased fatiguability [23]. An intrinsic muscular anomaly, instead of a decrease in skeletal muscle perfusion, is thought to be the cause of fatigue. [24]. There is a loss of muscle bulk, and a shift towards early anaerobic metabolism associated with the histological and enzymatic changes [25]. In support of this view, many of the observed abnormalities in the skeletal muscle structure and function observed in the subjects with heart disease are seen in normal controls with a similar level of inactivity [26]. However, a specific effect of heart disease, per se, is suggested by the failure of skeletal muscles to respond normally to exercise training in subjects with heart failure (HF) [27].

These changes are associated with both an increased ventilatory response to exercise and a reduction in exercise tolerance, with fatigue and breathlessness as prominent limiting symptoms [16,20]. Undoubtedly, the reduced cardiac output, impairment of pulmonary gas exchanges (e.g., restrictive syndrome due to thoracotomy), and impaired peripheral muscle function, due to the structural and metabolic abnormalities of skeletal musculature, decrease the muscular performance [5]. The contribution of the skeletal muscles to fatigue was due to a decreased blood perfusion; however, it was then shown that patients with cardiac disease and a normal blood flow also complained about exercise intolerance [28]. Thus, more important than the hemodynamic factors are the peripheral intrinsic changes.

In the present study, it is held that this performance is also affected by the reduced efficiency of the peripheral exercising muscles, which can be due to a decrease in blood flow.

The present study tests the hypothesis that peripheral muscular operating is also a limiting factor of exercise performance, which could play a role in dyspnea occurrence. To explore this hypothesis, the central and peripheral maximal and submaximal parameters of muscular exercise capacity were plotted against the dyspnea threshold, subjectively scored in children with a repaired congenital heart disease. The strength and endurance of the right knee muscle extensor have been measured to quantify the relationship between this threshold and the peripheral muscular factors.

## 2. Materials and Methods

### 2.1. Subjects

The approval of the experimental protocols was obtained from the Committee for Ethics in Research. Since humans were included in this study, a National Institute of Health (NIH) for protecting human research participants certificate was obtained. The potential risks of the study were explained to each child and their parents. The written informed consent was obtained from the parents, following the guidelines established by the institutional review board ethics committee. The experimental procedures complied with the ethical standards of the 1975 Helsinki Declaration.

A total of 13 children with congenital heart disease who had undergone surgical correction for congenital heart impairment (7 males and 6 females) and 14 healthy control children (8 males and 6 females) aged 13 ± 2 years were tested at the Cardio Pediatric department. All cardiac patient groups had undergone cardiac surgery reconstruction during infancy for congenital heart disease (before 2 years of age). Table 1 shows the characteristics of the patients and controls children. Seven subjects had pulmonary valve atresia, four had a transposition of great arteries, and two had tricuspid atresia. Throughout the research, the medication was not modified. Children that were infected did not take part in the study. No surgery or changes in heart function occurred in any of the people during the prior 24 months. Participants with unstable angina, aortic stenosis, acute myocardial infarction, obstructive cardiomyopathy, hypertension above 220/120 mmHg, or locomotor or mental disorders that could limit muscle performance were excluded from the study. Healthy subjects practicing sport for more than 3 h per week were excluded from the present study.

Weight was measured to the nearest 0.1 kg with a multi-frequency bioelectrical impedance analyzer (TBF-410GS, Tanita Co., Tokyo, Japan), and height was measured to the nearest 0.1 cm with a Harpenden stadiometer 602VR, with the child standing and wearing no shoes or heavy clothing.

The nutritional status was determined using the body mass index (BMI), which was then classified using the WHO Anthro and AnthroPlus software (Geneva, Switzerland).

The cardiac participants had undergone cardiac reconstruction surgery for congenital heart disease and expressed exercise limitations. They had the following specifications:

(1) Dyspnea with moderate exercises and belongs to class II of the functional categorization system of the New York Heart Association (NYHA);
(2) Absence of other disabling diseases;
(3) Ability to perform an exercise without an adverse cardiovascular disorder, such as arrhythmia. Subjects suffering from angina, aortic stenosis, a history of myocardial infarction, obstructive cardiomyopathy, or hypertension were not included in the study.

Medications were unchanged throughout the study, including converting the enzyme inhibitors, diuretics, cardiotonic, anticoagulant, and immunosuppressive drugs. Only, 2 (out of the 13) subjects were without drug treatment. Infected children were excluded from the research.

### 2.2. Protocol

A maximal exercise testing session on the ergo cycle and an assessment of peripheral muscular function on an isokinetic apparatus were randomly assigned to all subjects.

Based on the subject’s body mass, gender, and exercise habits, an individual and gradually incremental workload protocol was chosen [29], with gas exchange measurements in an air-conditioned room: 20 ± 1 °C: relative air humidity: 50%, air velocity lower than 0.2 m·s^−1^ (condition of free convection). All subjects were familiarized with the procedure and the equipment, before the experiment. Their clothing consisted of briefs, a tracksuit, cotton socks, and basketball boots for all the subjects. To rule out a possible effect of the circadian rhythm, tests were conducted at least 2 h following lunch and at the same time of day, usually 2 p.m.

On a separate day, a six-minute walking test and the lower limb strength were measured using a Cybex^®^ Norm II isokinetic dynamometer (Cybex-Lumex Inc, Ronkonkoma, NY, USA).

### 2.3. The Cardiopulmonary Exercise Test

Following the lung function measurements, the subjects were seated on the cycle ergometer while baseline measurements were taken. The subjects breathed through the apparatus, which was linked by the expiratory circuit to a breath-by-breath automated exercise metabolic system. The total dead space of the breathing apparatus (valve and pneumotachograph) was 120 mL, which was entered into the system software for correction. Cardiopulmonary exercise tests were performed in the upright position on a calibrated cycle ergometer (Ergoline 900). Each subject was thoroughly informed to maintain a cycling speed of 60 to 70 rpm throughout the testing, to refrain from speaking or standing on the pedals, and to continue pedaling until exhaustion. Subjects were instructed to breathe comfortably using a mouthpiece coupled to a pneumotachograph (Fleisch, Lausanne, Switzerland) equipped with a two-way low resistance breathing valve (0.9 cm H_2_O L^−1^ s, dead space of 50 mL, model 9340 occlusion valve, Hans Rudolph Inc, Kansas City, MO, USA). After 3 min baseline measurements, the subjects performed an incremental exercise. After completing three minutes of baseline measurements, the subjects engaged in an incremental exercise. Our laboratory’s personalized exercise testing approach often results in a (VO_2_ peak) test duration of 8 to 12 min, which is consistent with classical exercise testing recommendations [29]. All subjects were encouraged to continue exercising until dyspnea or fatigue forced them to stop. Before each test, gas and volume calibrations and the lag time of the analyzer were carefully checked. The VO_2_ and VCO_2_ measured by the breath-by-breath systems were normalized for conventional ambient temperature and 760 mmHg pressure (STPD). Following each test, the cardiorespiratory follow-up was prolonged over a 5 min recovery period.

All subjects were invited to exercise until they reached the point of exhaustion or felt unable to continue. When three of the following conditions were met, the exercise was considered maximal [19,29]: (i) exhaustion of the subject or inability to maintain the required pedaling frequency (>50 rev/min) despite strong verbal encouragement; (ii) exertional dyspnea or leg pain; (iii) VO_2_ plateau was attained (the VO_2_ plateau was was defined as the point at which the final increase in the VO_2_ did not exceed VO_2_ < 5 mL·min^−1^ for a 5–10% increase in effort) [19]. The peak VO_2_ was described as the highest VO_2_ elicited during the exercise test in the absence of if a VO_2_ plateau, and we considered the predicted VO_2_ peak to have been reached if the VO_2_ peak value recorded was greater than 85% of the predicted value); (iv) predicted maximum HR (HRmax) attained (210 − 0.65 × age]) ± 10%) (We considered the predicted HRmax to be obtained if the observed HR was higher than 90% of the predicted value.); and (v) maximal rate of exchange ratio (RERmax) of > 1.15 [30].

The minute ventilation (VE), respiration rate (f), oxygen consumption (VO_2_), and carbon dioxide production (VCO_2_) were continuously recorded. The heart rate (HR) was recorded for 1 min at rest, 5 min of the warm-up, and throughout the exercise period. Twelve ECG derivations were monitored during the exercise and recovery periods. A sphygmometer was used to measure the blood pressure on the upper right arm during the exercise (Quinton).

The maximal exercise can assess functional capacity but, it can be influenced by the motivation of the children [31]. To avoid this problem, the ventilatory threshold (VTh) was scored as an alternative method to quantify the exercise capacity (Wasserman, 1997) and determined, as previously described [19,29]. Three validated methods were applied concurrently to estimate the VTh from the incremental exercise test data: the ventilatory equivalent method (VE/VO_2_ method) [32], (2) excess carbon dioxide method (PETCO_2_) [33], and (3) modified V slope method [34]. This value was determined using a double-blind method, based on highest degree of agreement made between two independent observers. As there was disagreement (i.e., a difference of more than 10% between the two observers), a third observer was invited to assess the thresholds. The value maintained was the mean of the values that were more closely agreed upon. The ventilatory threshold was defined as the work rate that was most consistent across the various approaches for determining the ventilatory threshold.

The definition of “dyspnea” was carefully explained before exercise, according to a standardized procedure consistently given by the same observer and defined as having an unpleasant sensation or uncomfortable breathing. Care was taken to explain the difference between leg pain and breathlessness [15]. A visual analog scale (VAS) was used to rate dyspnea at rest, during the warm-up period and during the last 15 s of each exercise step, equipped with 40 diodes. The VAS’s left and right ends were labeled “no dyspnea” and “maximal dyspnea”, respectively. The light signal was controlled by an interrupter located on the handlebar. Blind to the subject, the numerical values corresponding to the dyspnea score ranged from 0 (no dyspnea) to 10 (maximal dyspnea).

### 2.4. Six Minute Walk Test

Many cardiac rehabilitation programs now include submaximal exercise testing with the six-minute walk test (6MWT) for initial evaluation and to establish functional outcomes after cardiac rehabilitation program. The 6MWT test was originally described by Guyatt et al [35]. It is straightforward to administer, has high patient acceptance due to the fact that the exercise levels during the test closely approach those of the patient’s daily activities, and has low administration costs. The 6MWT has been commonly deployed in the research setting as a measure of functional capacity and an indicator for disease severity and prognosis in subjects with cardiac and pulmonary disease. The 6MWT was performed in a covered corridor of 30 m in length that was marked every 2 m on a plane surface. The tests were carried out in accordance with the recommendations of the American Thoracic Society [36]. Subjects were advised to cover the maximal distance in the allotted time. The participants were permitted to pause and rest throughout the test, but were asked to begin walking immediately upon feeling ready. According to Guyatt et al. [35], the children received standardized encouragements. For all the subjects, the 6MWT was monitored by the same person. At every minute, the participants were informed of the remaining time of the test. The total distance walked was retained at the end of the test [36].

### 2.5. Peripheral Skeletal Muscle Function Assessment

Isokinetic dynamometry measures the joint moment exerted throughout continual joint angular velocity movements to assess dynamic muscle strength. It is widely used in sport, exercise, and pathological conditions to determine muscle strength; to evaluate training or rehabilitation programs; to predict performance; to prevent injuries; and to conduct fundamental research on the mechanics of muscles, tendons, and joints, modeling and simulation [37,38].

Muscular functions are evaluated by measuring the force and fatigue generated during muscular contractions [39]. Muscle fatigue is defined as the inability of a muscle to maintain a certain degree of contraction force [39,40,41]. The right knee extensor muscle strength and endurance were determined using an isokinetic dynamometer (A Cybex^®^ Norm II System, Sugar Land, TX, USA) with a designed computer program, including compensation for the torque due to the masses of the lower limb and the lever of the dynamometer. Before starting each test, the torque and angular velocity were calibrated. The limb position and torque correction for height and body mass were adjusted just before each movement.

The maximum voluntary isometric force (MVIF) was used to assess skeletal muscle performance and the muscle endurance corresponded to the time to exhaustion or limit time (Tlim), at a considerable intensity level of the percentage (%) of the maximal voluntary isometric force. The subjects were sitting with their hips at a 90° angle. The right lower leg was fixed to the lever arm of the dynamometer. The thorax and the pelvis were strapped to limit the movements of the other osteoarticular chains. The thigh was attached in place above the knee. The pivot point of the lever arm was aligned with the knee joint. The measures were performed on the right leg.

The maximal muscle strength in the right knee extensor muscle was recorded as the torque output during the maximal voluntary knee extension with the constant angular velocity at 60 degrees·s^−1^. Subjects had to push 3 times against the lower leg with the maximal voluntary knee extension. A 2 min recovery period was allowed between each maneuver. Each subject could visualize the generation of their muscle force on a computer screen while developing the maximal voluntary isometric force; also, encouragement was delivered to the participants during this maximal test to reach the best results. The peak values of each test were automatically detected and averaged over the three tests.

In order to determine the time to exhaustion, the patients were asked to hold an isometric contraction for as long as they could, at 50% of their maximal voluntary isometric power. The time to exhaustion test causes skeletal muscle fatigue because our skeletal muscles have a limited blood supply, and this test primarily requires anaerobic metabolism. A visual reference mark was placed on the computer screen corresponding to 50% of the maximal voluntary isometric force, which the patients could manage as best as they could. The maximal voluntary isometric force values were expressed in N·m^−1^ for strength and in sec for static endurance time.

### 2.6. Dyspnea Measurements

The visual analog scale (VAS) is a continuous scale represented by a vertical or horizontal line that is typically 100 mm long [15,42]. At the VAS’s endpoints, anchors such as “no breathlessness” and “maximum breathlessness” might be placed. The patient was told to make a mark on the VAS or to modify a cursor visible on a computer screen to indicate the degree of breathlessness. The dyspnea score/VO_2_ relationship identified a dyspnea threshold as the point at which the dyspnea score increased suddenly. This value was determined via exponential regression analysis.

### 2.7. Data Analysis

For each subject, the maximal values of VO_2_, HR, VCO_2_, and VE, and the power output P were measured from the cardiopulmonary exercise test as the average maximal value recorded over a 30 s period. Subsequently, the VO_2_ peak (mL·kg^−1^·min^−1^), the work efficiency VO_2_/P (mL·min^−1^w^−1^), the oxygen pulse VO_2_/HR (mL·kg^−1^·beat^−1^), respiratory gas exchange ratio (RER), ventilatory equivalent for O_2_ (VE/VO_2_), and ventilatory equivalent for CO_2_ (VE/VCO_2_) were calculated.

### 2.8. Statistical Analysis

The values are presented as the mean ± standard deviation (SD). A 2-tailed test, a medium effect size (d = 0.50), and an alpha of 0.05 were used in an a priori power analysis using G*Power3 [43] to evaluate the difference between two independent group means. A total sample of 27 patients with 13 CHD and 14 healthy subjects was necessary to attain a power of 0.80, according to the findings. Cohen’s effect sizes (d) were estimated using data on mean differences, number of individuals, and mean pooled standard deviations (SD), and were classified as small (0.20), moderate (0.50), and large (0.80) [44]. The differences between the two groups were compared using an unpaired Student’s test or the Mann–Whitney test, when both the normality (Kolmogorov–Smirnov) and equality of the variance (Levene’s median test) test failed.

The coefficient of correlation between the values is presented as mean ± SD. Analyses were carried out using SPSS software (IBM Corp. Released 2016. IBM SPSS Statistics for Windows, Version 25.0, Armonk, NY: IBM Corp). A *p*-value < 0.05 was considered as significant.

## 3. Results

The anthropometric characteristics of the CHD subjects and controls are presented in Table 1. There were no significant differences between the groups in age, weight, height, and BMI.

The maximal power output, oxygen consumption, heart rate, pulmonary ventilation, oxygen pulse, and energetic cost are presented in Table 2.

**Figure 1:**
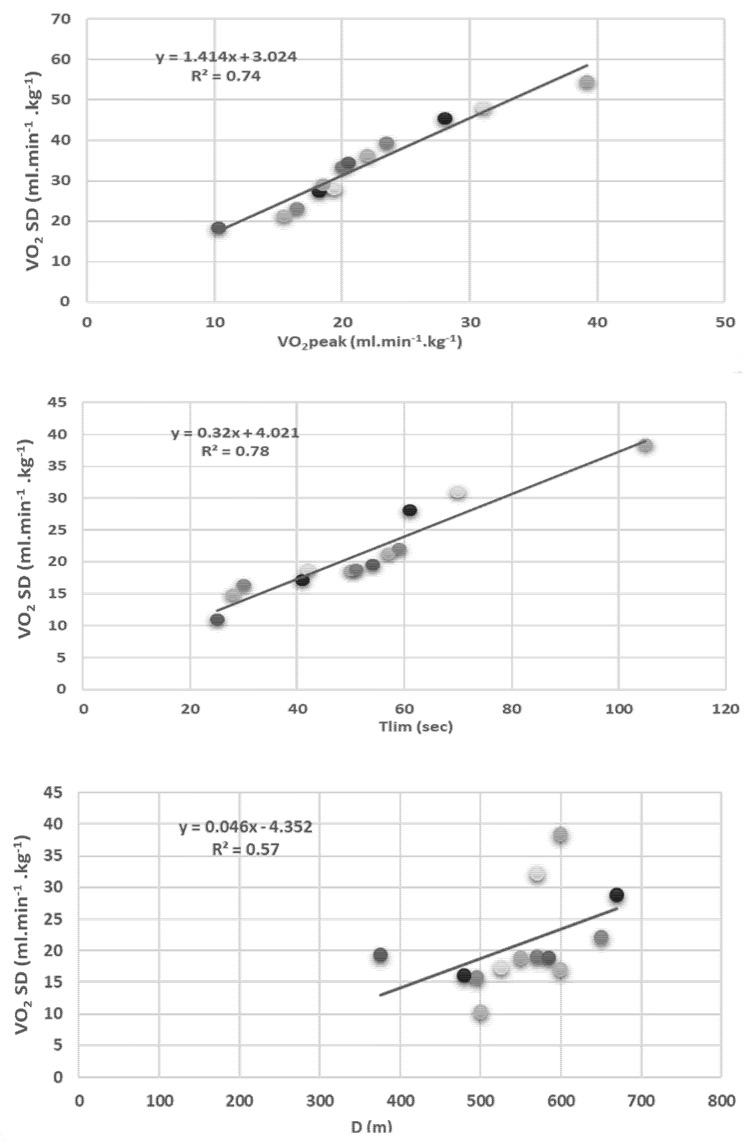
Relationship between oxygen consumption measured at the dyspnea threshold (VO_2_ DT; mL·kg^−1^·min^−1^) and the peak oxygen consumption (VO_2_ peak; mL·kg^−1^·min^−1^), time to exhaustion (Tlim; sec) at 50% of maximal voluntary isometric force measured on isokinetic cybex, and the distance walked (D;m) during a 6 min walk test in CHD patient.

The average maximal measured in the patients’ values was lower than the controls for the relative VO_2_ (*p* < 0.001), HR (*p* < 0.001), power output (*p* < 0.001), and minute ventilation (*p* < 0.001) (Table 2). The relationships between the oxygen consumption measured at the dyspnea threshold (VO_2_DT, mL·kg^−1^·min^−1^), time to exhaustion duration (Tlim) (sec) of 50% maximal voluntary contraction, and the distance walked d (m) during the 6 min walk test are shown in Figure 1.

As shown in Figure 1, the dyspnea threshold of the cardiac subjects was significantly related to the VO_2_ peak (R^2^ = 0.74; *p* < 0.01), the time to exhaustion (R^2^ = 0.78; *p* < 0.01), and the distance achieved during the 6MWT test (R^2^ = 0.57; *p* < 0.05).

The submaximal cardiorespiratory parameters HR, VO_2_VTh, power output, minute ventilation, and VO_2_/P were significantly decreased in cardiac subjects. No other effect was found (Table 3).

As regards the maximal peripheral muscular parameters, the muscle strength was decreased in the cardiac subjects (Table 4). The intergroup muscle endurance differences tended to reach a significant level (*p* = 0.05).

## 4. Discussion

The purpose of this study was to assess cardiorespiratory and peripheral muscle function, as well as their relationship with the subjective dyspnea threshold in children after surgical correction for congenital heart disease.

The main finding of our study was that the dyspnea threshold was significantly related to the VO_2_ peak, time to exhaustion, and distance achieved during the 6 min walk test. Our findings show that VTh confirms this recommendation because, at the ventilatory threshold, the heart rate and power output were associated with a reduction in the muscular capacities following surgery.

VO_2_ peak, the gold standard for the assessment of exercise tolerance, is described as “the maximal capacity of the cardiovascular system to transport oxygen to exercising skeletal muscle and the maximum capacity of exercising muscle to extract oxygen from the blood” [45]. Our results demonstrate reductions in VO_2_ peak and exercise tolerance [46]. The possible reasons for a decreased VO_2_ peak were complex. Deconditioning, a failing rise in pulmonary blood flow during exercise, a rising systemic shunt volume, and increased hypoxemia may contribute to decreased respiratory gas exchange, VO_2_ peak, and exercise capacity [8]. Additionally, there are considerations about reduced regular activity as a result of parental overprotection, which reduces the patients’ ability with CHD to enhance their exercise capacity.

The exercise-induced heart rate response is a complicated mixture of the chronotropic reserve during exercise and the post-exercise heart rate recovery. When gender, age, and body mass index are all controlled, multivariate analysis reveals that the VO_2_ peak is associated with maximal heart rate [47]. Our data suggest that chronotropic incompetence has a crucial part in explaining the lower work exercise capacity of subjects with CHD, with maximal heart rates at about 30 bpm lower than in the normal subjects. CHD patients have an insufficient chronotropic response indicated by the failure to elevate the HR in response to metabolic demand [48]. This leads to a lower maximal HR, because autonomic dysfunction, ischemia and/or denervation, and cardiac arrhythmias are common in people with congenital heart disease, even after surgical repair [8].

Oxygen pulse is a good indicator of cardiovascular function in the pediatric population, and can provide additional information on the predictive significance of exercise capacity related to cardiovascular disease [49]. It is a measurement of stroke volume and peripheral oxygen extraction during exercise, which reflects myocardial oxygen delivery and cardiac functional reserve under physiological stress [9]. In our study, the O_2_ pulse increased normally with incremental exercise. The basic shape of the O_2_ pulse appears to be hyperbolic over the range in which VO_2_ increases linearly with HR, with a quick rise at low work rates followed by a gradual approach to the asymptotic value [50]. As described by Mantegazza et al [8], the O_2_ pulse must be interpreted with caution and in conjunction with the heart rate. For the same reasons, work efficiency (VO_2_/P), which is an indicator for metabolic cost of external exertion, could be impaired in patients with congestive heart failure [51].

Additionally, a decrease in heart rate during peak exercise was identified as a factor contributing to the decreased VO_2_ peak. However, the time-dependent decrease in peak exercise heart rate was less apparent in this series than the time-dependent fall in peak exercise O_2_ pulse. These findings highlight the negative effects that an impaired chronotropic response can have on exercise performance in people with CHD [52]. 

In CHF, the VO_2_ peak is proportional to the peak exercise cardiac output and muscle blood flow. However, failure to raise cardiac output correctly results in an inadequate raise in perfusion to the exercising muscle, which can result in rapid anaerobic glycolysis, muscular fatigue, and eventually muscle wasting [25]. 

As a consequence of dyspnea feeling, most patients with CHD decreased their efforts during exercise to prevent the dyspnea perception. Consequently, anaerobic metabolism became prevalent. The anaerobic threshold (AT) index, which is used to determine exercise capacity, is a measure of the transition from aerobic to aerobic plus anaerobic metabolism [53]. It represents the cardiovascular system’s capacity to meet the rising energy demand of exercise. and to differentiate noncardiac (pulmonary or musculoskeletal) against cardiac factors of exercise limitation, considering patients who fatigue prior to VT are likely to have a noncardiac disease [54]. In our study, the AT is below normal [55], and we hypothesized that the VT may be more accurate than the VO2 peak due to a lower sensitivity to error.

CHF is characterized with exercise intolerance as a result of dyspnea and fatigue. Traditionally, these symptoms were assumed to be exclusively due to central hemodynamics. However, it is now recognized that CHF has also significant skeletal muscle pathology, which could contribute to exercise intolerance. Several studies have found poor correlations between exercise tolerance and the measurements of cardiac function in subjects with congestive heart failure [56,57]. Our results demonstrate muscle dysfunction, including muscle weakness, and a decreased and increased reliance on anaerobic glycolysis. Muscle dysfunction, as described by peripheral circulation abnormalities, and muscle atrophy can contribute to the reduced exercise tolerance observed [58], and physical deconditioning appears to play a contributory role [59]. In support of this view, many of the observed abnormalities in skeletal muscle structure and function observed in patients with CHF are seen in normal controls with a similar level of inactivity [60].

During graded maximal exercise with the lower limbs, normal subjects recruit at least 50% of their total skeletal muscle mass, exhausting their cardiopulmonary reserve. When compared to normal individuals of comparable age, subjects with CHF exhibit a lower limb (LL) skeletal muscle mass that interacts with the degree of exercise intolerance [58]. Our results demonstrate a great correlation between the maximal oxygen uptake and time to exhaustion. It is generally known that, with congestive heart failure, the maximal voluntary isometric force diminishes and is reliant on the muscle size and motor unit recruitment [61]. According to the results obtained for the maximal voluntary isometric force, the capacity of congestive heart failure to develop maximum force has been reduced [62]. Subjects with congestive heart failure, on the other hand, had less quadricep exhaustion time than the healthy subjects, indicating greater skeletal muscle fatigue. Because skeletal muscle blood flow is restricted during the time to exhaustion test, skeletal muscle fatigue occurs, and this test primarily requires anaerobic skeletal muscle metabolism. Skeletal muscle fatigue is caused by changes in the muscular energy metabolism, which are unrelated to the decrease in local blood flow during isometric exercise [56].

Breathlessness is the cardinal symptom of heart failure. Its significance is emphasized by the fact that dyspnea severity is used to determine the New York Heart Association’s functional classification for heart failure. A relationship was found between the VO_2_ dyspnea threshold (VO_2_ DT) and VO_2_ VTh [63]. Moreover, significant correlation coefficients with lower values for the standard error of the estimate were found. Even though the physiopathologic mechanisms going with dyspnea perception are not well known, literature studies are concordant to link dyspnea sensation during exercise to ventilatory increase [64]. Indeed, dyspnea and ventilatory thresholds were reported to be under the same command by Rampulla et al. [65]. In our subjects, the perceived efforts of exercising the muscles and breathing for a given power output are greater than in normal subjects of the same age and stature. These changes are not solely due to the impaired circulatory responses to exercise. Weaker muscles, which have a reduced aerobic metabolic capacity, lead to an increase in perceived effort that is inversely proportional to the strength of the muscles.

## 5. Study Limitations

The lack of randomization and a small sample size limit this study’s conclusions. The physical activity level was not evaluated in this study, and we can only assume from the CPET and the 6 min walk test that the mechanism by which the study’s findings were obtained cannot be identified because the cardiac output, systemic oxygen delivery, leg muscle blood flow and oxygen delivery, and muscle mass morphological and structural evaluations were not evaluated.

## 6. Conclusions

In conclusion, the present study shows that not only the cardiac output but also peripheral muscle performance can be considered as limiting factors of exercise capacity after corrected congenital heart disease in children. The dyspnea threshold can characterize the parameters of exercise capacity in these children.

Dyspnea and ventilatory the threshold appear to be highly correlated. Between these two parameters, a significant association with good correlation coefficients and perfect agreement was obtained. Thus, the dyspnea threshold can be utilized to evaluate cardiorespiratory fitness in infants with congenital heart disease. Additionally, this index has significant real advantages in the rehabilitation center; it is inexpensive, convenient for using, and produces speedy results. However, additional research should be undertaken to investigate whether training affects the relationship and correspondence between dyspnea and ventilatory gas exchange thresholds found in this study. The assessment of these children from a non-invasive method can be used in the field of cardiac rehabilitation to replace more complex methods.

## Figures and Tables

**Table 1:** Characteristics of children with congenital heart disease (CHD) and control subjects.

	Patients	Controls	*p*-Value
n	13	14	
Anthropometric data			
Age (year)	14 ± 1	13 ± 2	n. s.
Body mass (kg)	53 ± 11	51.2 ± 8.7	n. s.
Height (cm)	162 ± 8	160.1 ± 8.8	n. s.
BMI (kg^−2^)	20.3 ± 0.7	19.8 ± 1.8	n. s.
Diagnosis			
PVA	7		
TGA	4		
TA	2		

Definition of abbreviations: values are mean ± standard deviation. PVA = pulmonary valve atresia, TGA= transposition of great arteries, and TA = Tricuspid atresia.

**Table 2:** Maximal cardiorespiratory variables.

	Patients	Controls	*T* test
VO_2_ (mL·kg^−1^min^−1^)	33.8 ± 8.9	46.7 ± 6.7	*p* < 0.001
HR (beat.min^−1^)	174 ± 9	201 ± 10	*p* < 0.001
VO_2_/P (mL·min^−1^w^−1^)	11.97 ± 2.37	11.2 ± 1.7	NS
Power output (W)	117 ± 27	223 ± 29	*p* < 0.001
VE (L·min^−1^)	65.68 ± 15.91	106.4 ± 24.6	*p* < 0.001
VO_2_/HR (mL·min^−1^·beat^−1^)	11.13 ± 0.59	11.32 ± 0.43	NS

Definition of abbreviations: the values are mean ± standard deviation. VO_2_ = oxygen uptake, HR= heart rate, VO_2_/P = work efficiency, P =Power output, VE = minute ventilation, and VO_2_/HR = Oxygen pulse. The *p*-values are indicated when the f-values were significant. The f-values are given with their corresponding degrees of freedom (subscripts beneath the f-values). A: measured maximal values; B: theoretical maximal values; and NS: non-significant. Differences were considered significant for *p* < 0.05.

**Table 3:** Sub maximal parameters of the exercise capacity scored at ventilatory thresholds.

	Patients	Controls	*p*-Value
VO_2_ (mL·kg^−1^·min^−1^)	19.2 ± 5.2	32.36 ± 6	*p* < 0.001
HR (beat·min^−1^)	116 ± 11	164 ± 10	*p* < 0.001
P (W)	48 ± 14	72 ± 21	*p* < 0.01
VE (mL·min^−1^)	25.0 ± 6.2	36.4 ± 5	*p* < 0.001
VO_2_/HR (mL·kg^−1^·beat^−1^)	8.9 ± 4.6	9.34 ± 5	NS
VO_2_/P (mL.kg^−1^·W^−1^)	13.52 ± 3.72	12,20 ± 3.5	*p* < 0.001

Definition of abbreviations: values are mean ± standard deviation. VO_2_ = oxygen uptake, HR= heart rate, P =Power output, VE = minute ventilation, VO_2_/HR = Oxygen pulse, and VO_2_/P = work efficiency. Differences were considered significant for *p* < 0.05.

**Table 4:** Muscular strength and endurance time.

	Patients	Controls	*p*-Value
MVC (N/m)	120.8 ± 41.9	186.26 ± 39.59	*p* < 0.001
Tlim (Sec)	53 ± 21.4	67.64 ± 14.77	*p* < 0.001

Definition of abbreviations: values are mean ± standard deviation. MVC = maximal voluntary contraction and Tlim = limit time to exhaustion at 50% maximal voluntary contraction.
